# Factors Associated with Musculoskeletal Disorders among Regular and Special Education Teachers: A Narrative Review

**DOI:** 10.3390/ijerph191811704

**Published:** 2022-09-16

**Authors:** Ahmad Asyraf Abdul Rahim, Mohammad Saffree Jeffree, Dayang Maryama Ag Daud, Nicholas Pang, Mohd Fazeli Sazali

**Affiliations:** Faculty of Medicine and Health Sciences, Universiti Malaysia Sabah, Jalan UMS, Kota Kinabalu 88400, Sabah, Malaysia

**Keywords:** musculoskeletal disorders, special education teachers, ergonomics, designed workplaces

## Abstract

Musculoskeletal disorder (MSD) is a major health problem, which can lead to an enormous burden to the institution as well as chronic disability to the individual. Teachers are at risk of developing MSD due to the exposure to various ergonomic risk factors. Teachers of special education, for example, are expected to perform extra duty such as lifting and moving students, feeding food, changing diapers, and helping them in ambulation. Although there is an adequate amount of scientific research on MSD’s prevalence and its risk factors among regular teachers, only few studies have focused on special education teachers. This review aimed to address these gaps by describing the evidence from various papers on the prevalence of MSD among regular and special education teachers and the related risk factors. The papers have been gathered using electronic databases, including PubMed, Science Direct, Google Scholar, and Springer. The prevalence of MSD among regular teachers ranges from 48.7% to 73.7%, while the prevalence ranges from 38.7% to 94% in special education teachers. Risk factors, such as individual (age, duration of teaching, working hours, and work burden), physical (teaching activities, affected body areas), and psychological factors (stress, anxiety, fear), were identified. From the review, it is recommended to implement ergonomically designed workplaces, comprehensive ergonomic training, psychological approaches, and functional training among teachers at risk.

## 1. Introduction

Musculoskeletal disorders (MSD) are a widespread occupational issue in the teaching profession, and teachers tend to have a high prevalence of MSD [1,2]. It is a massive burden on the institution, with expenses such as sick pay, missed productivity, retraining, legal fees, and injury benefits adding up quickly [3]. Musculoskeletal disorders, often known as “ergonomic injuries”, occur when the body’s muscles, ligaments, and tendons are used to perform tasks, often in awkward positions or repetitive tasks, resulting in discomfort and disability over time. MSD is most often suffered by teachers in the shoulder, lower back, knee, and wrist, which results in teachers most often suffering from shoulder tendinitis, intervertebral disc prolapses, knee osteoarthritis, and carpal tunnel syndrome [4,5]. Symptoms can range from mild and intermittent to extreme, chronic, and debilitating conditions and can include pain, swelling, numbness, and tingling in the affected region and joint movement restriction [6].

Ergonomics is a scientific field that focuses on understanding the interactions between humans and other system components. The ergonomics profession applies theory, concepts, data, and methodologies to design to maximize human well-being and effective system performance [7]. For example, repetitive strain injuries and other MSD problems, which can develop over time and result in long-term incapacity, must be avoided by proper ergonomic design. Ergonomists examine the work at hand and the demands placed on the user, the tools at hand (their size, shape, and suitability for the activity), and the information at hand to determine if a person and the technology being used are a good fit (how it is presented, accessed, and changed). Various advanced tools and mathematical modeling have been created to apply the ergonomics concept in work, including risk exposure monitoring, lean tools, and intervention [8,9,10].

Although teachers are putting forth great effort to educate our children, specific work-related health issues have gone unnoticed. The problem is that musculoskeletal disorder cases among teachers are high, especially those working in special education schools or programs. This issue could be seen in a study conducted in Taiwan, which revealed that 94% of special education teachers have musculoskeletal disorders [11]. A special education teacher is defined as working with students with a wide range of learning, physical, mental, and emotional disabilities. Regular teachers’ job tasks can include a lot of sustained standing and head-down postures, such as during reading, assignment correction, and board writing [12]. When dealing with students with disabilities, special education teachers have an additional set of tasks and curricula compared to regular teachers; they also provide nursing care such as lifting and moving students, feeding food, changing diapers, and helping them in ambulation [13]. If this activity is not conducted correctly, it will increase the risk of musculoskeletal disorders.

Since the real cause of the ergonomic problem was difficult to identify, the musculoskeletal symptoms that developed have affected the quality of work, and teachers had to take an extended sick leave. According to a systematic study, musculoskeletal disorders in teachers are linked to various factors, including individual characteristics, such as gender and duration of service, physical factors, such as teaching posture, and psychological factors, such as stress [4]. According to Malaysian studies, stress has a significant role in musculoskeletal disorders among teachers [14,15]. Despite their potential for occupational health issues, the difference in the prevalence of MSD among regular and special education teachers is still limited. In addition, studies on MSD risk factors are still lacking, especially the teaching activities at risk of causing MSD in particular body parts among regular and special education teachers. Therefore, it is crucial to look at teachers’ risk factors for MSD, namely individual, physical, and psychological factors.

Thus, this review aims to analyze the literature and report on the prevalence of MSD and possible associated risk factors among regular and special education teachers. The study focused on primary, secondary, and special education school teachers.

## 2. Materials and Methods

A literature review, empirical research, and case studies from peer-reviewed English publications were considered for inclusion, while letters to the Editor and conference proceedings were excluded. Articles and research meeting the following criteria were included: full-time teachers, more than one year of teaching experience, risk factors associated with MSD, and prevalence of MSD in the upper and lower limbs. The exclusion criteria were: articles for which full text was not available, were not in English, or were grey literature. There were no age, gender, ethnicity, or socioeconomic status restrictions.

Studies on MSD among regular and special education school teachers have been gathered using electronic databases, including PubMed, Science Direct, Google Scholar, and Springer. The search terms used were: “school teachers”, “special education teachers”, “musculoskeletal disorders”, “work-related musculoskeletal disorders”, and “psychological stress”. The names, keywords, and abstracts of every research item found during the search were examined for potential relevance to this narrative review. Full-text copies were obtained for analysis and data extraction for all papers that fulfilled the inclusion requirements.

## 3. The Prevalence of MSD

The number of teachers with MSD is increasing due to teachers working tirelessly to educate our students, and this problem is often underestimated [4]. Based on studies conducted in several countries, the country that recorded the highest MSD among regular teachers was Egypt, having the highest prevalence (76%), followed by Thailand (73.7%), Brazil (73.5%), and Malaysia (72.9%) (Table 1) [16,17,18,19]. In addition, a study in Africa involving 1747 teachers reported that 55.7% of them suffered from lower back pain. It also reported that lower back pain caused minimal disability (67.1%), moderate disability (27.9%), and severe disability (4.3%), with 0.7% of them crippled. [20]. Meanwhile, studies in Ethiopia and China stated that MSD occurred in 57.3% and 48.7% of the teachers, commonly at the neck and shoulder [21,22].

Special education teachers have a different additional set of tasks and curriculum than regular teachers when teaching students with disabilities such as visually impaired, hearing problems, intellectual disability, learning disorders, autism, or other neurodevelopmental disorders [23]. A study in Taiwan among early intervention teachers who served special needs students reported the highest prevalence of MSD (94%), mostly involving the shoulder region, followed by the lower back and neck (Table 2) [11]. In 2016, another study was done in Taiwan among special education teachers and reported a lower prevalence of MSD (86%), which most commonly affects the lower back, shoulder, and wrist. The study also reported that MSD interfered with work among 80% of teachers, and 17.4% had to take sick leave [13]. Physical and mental problems are common among special needs students, and many of them cannot manage and adapt to their surroundings daily [24]. This adds to the teacher’s workload, because they teach and offer nursing care, such as lifting and transferring students, feeding them, changing diapers, and assisting them with ambulation, among other things [12]. These facts were proven in a study involving special education teachers in Italy, which showed that 85.9% of special education teachers had lower back pain and 46.5% had neck pain [25].

In Malaysia, a study done among the special education teachers in Kelantan showed that 72% of the teachers suffered from MSD, especially in the lower back, shoulder, and neck areas [26]. Meanwhile, Hong Kong, Japan, and Germany reported lower prevalence of MSD, with 55%, 45%, and 38.7% of their special education teachers having back pain, respectively [24,27,28]. Although there are not as many studies conducted on special education teachers as regular teachers, the current prevalence reported is worrying. If the proper initial steps could be taken, then the problem of MSD among special education teachers will be reduced.

## 4. Individual Risk Factors

### 4.1. Effect of Age

While age has been significantly associated with MSD, the findings of the study are mixed, with some studies claiming that older teachers are more likely to develop MSD, and other studies have reported that younger teachers are more significant [4,29]. For example, in Germany, a study said that an increase in age would increase the prevalence of MSD among special education teachers (AOR = 1.03, 95% CI = 1.00–1.05) [28]. The most probable cause for the increased frequency of MSD among older teachers is that as people age, their muscle mass declines, they lose connective tissue flexibility, and the cartilage between their joints thins [30]. Furthermore, tissue repair slows as people become older, as the body deals with a lifetime’s worth of soft tissue injury. However, studies have also proven that older-aged teachers had a lower prevalence of MSD. Supporting this hypothesis are the results from a Taiwan study, where special education teachers above 40 years old were less likely to develop neck pain (OR = 0.321, 95% CI = 0.159–0.649) and lower back pain (OR = 0.347, 95% CI = 0.172–0.703) than younger teachers [11]. Atikah et al. also reported a similar finding in their study, where teachers below 40 years old had a higher prevalence of MSD than teachers aged 40 and 50 years old [26]. The explanation for this might be that teachers with severe MSD symptoms may have already left their careers; thus, the more experienced ones who stayed on were more likely to have fewer work-related MSD issues. Another factor might be that teachers over the age of 40 are better aware of job-related health concerns and less likely to be harmed [11].

### 4.2. Duration of Teaching Experience

The duration of teaching experience was significantly associated with MSD among special education teachers. According to H. K. Cheng et al., teachers with experience of more than three years were two times more likely to develop MSD than less experienced teachers (OR = 2.782, 95% CI = 1.244–6.218) [11]. The longer someone is exposed to occupational risk factors, the more likely they are to develop work-related disorders or injuries. Meanwhile, special education teachers with more than five years of experience were four times more likely to have MSD compared to teachers who worked for a shorter period, as reported in another study in Taiwan (OR = 4.090, 95% CI = 1.350–12.390) and supported by a study in Kelantan [13,26]. This may be due to experienced teachers being generally allocated to more challenging students, and as a result of their frequent burdensome responsibilities, microtrauma would develop over time [13].

### 4.3. Working Hours and Nap Time

Long weekly working hours exposed teachers to conditions that have been associated with MSD, such as prolonged standing, prolonged sitting, or awkward posture. Working hours have also been shown to have a direct effect on emotional fatigue and a significant impact on MSD and the dimensions of professional effectiveness and cynicism, according to studies [31,32]. For example, in Taiwan, special education teachers working more than five days per week had a four-fold higher risk of developing MSD than teachers working fewer than five days per week (OR = 4.256, 95% CI = 1.158–15.642) [11]. Furthermore, teachers who had no time for a nap during office hours also experienced physical and mental fatigue, besides prolonged working hours. This problem was discovered by Atikah et al., who showed that special education teachers who did not spend time napping during working hours had a significantly higher prevalence of MSD (*p* < 0.001) [26]. In contrast, special education teachers in Taiwan who practiced napping during office hours were less likely to develop MSD (OR = 0.442, 95% CI = 0.230–0.851) [13]. According to Da Costa and Vieira, individuals who napped during work hours had fewer physical demands placed on them, which allowed their bodies to heal [33].

### 4.4. Teaching Burden and Partner

Generally, teachers with a more significant burden of teaching and nursing responsibilities on students with special needs are more likely to develop MSD. It has been proven in a study in Kelantan reported that a higher number of students has significantly increased the prevalence of MSD, and this is worsened by the absence of teaching partners in class (*p* = 0.046) [26]. Moreover, students with multiple impairments are extremely reliant and have a significant association with MSD. A study in Taiwan reported that students with multiple disorders increase the risk of MSD among teachers by more than two-fold compared to single disorder students (OR = 2.412, 95% CI = 1.100–5.287) [13]. Teachers had to serve appropriate assistance to those students in their daily tasks, such as diapering, eating, toileting, transferring, and rehabilitation. However, teaching partners might be helpful because they could share their burdens in managing children with disabilities, reducing the likelihood of MSD occurrence. This was proven by H. K. Cheng et al. in their studies that by having partners during a teaching in class, teachers would be less likely to develop MSD (OR = 0.387, 95% CI = 0.159–0.941) [11].

## 5. Physical Risk Factors

### 5.1. Regular Teaching Activities

Ergonomics risk factors are elements of work or activities that put individuals’ bodies under biomechanical stress, potentially leading to ergonomics-related diseases or injuries [34]. Awkward postures, static and sustained work postures, forceful exertion, and repetition are among them, and they pose a risk to the teachers in their everyday job. For example, Temesgen et al. stated that regular teaching activities involving static head down posture (OR = 2.26, 95% CI = 1.55–3.33), elevated arm over shoulder (OR = 2.71, 95% CI = 1.86–3.95), and prolonged sitting (OR = 1.50, 95% CI = 1.02–2.23) were more likely to develop MSD among the teachers [22]. Meanwhile, in Africa and Thailand, awkward arm (OR = 1.81, 95% CI = 1.24–2.62) and body posture (OR = 1.7, 95% CI = 1.06–1.70), especially during writing on the board, were significantly related to MSD [18,20].

### 5.2. Special Education Teaching Activities

It is tough to teach physically and mentally impaired students. Unlike regular teachers, special education teachers spend a significant part of their workdays on jobs that need them to move and posture in ways that stress their bodies. In Hong Kong, a study revealed that teachers with MSD spent a significantly longer time in static trunk posture (*p* < 0.05) and trunk flexion for more than 10° (*p* < 0.0125) when compared to regular teachers [24]. This can be observed when helping the student in ambulation and performing activities. Special education teachers are regularly asked to lift and carry students, aid them in positioning, transfer students from one location to another, change diapers, feed, pull and push, and stand for lengthy periods. In Taiwan, special education teachers who did not involve in diaper changing, feeding, toileting, grooming, transferring, rehabilitation, and getting in and out of a vehicle were proven to be less likely to develop MSD than those teachers who were involved with the tasks (*p* < 0.01) [13].

Furthermore, students identified as having multiple impairments that needed multidimensional attention and had difficulty taking care of themselves, increasing the teacher’s effort and risk of MSD. This has been proven by Atikah et al. that assisting in diaper changing (*p* = 0.011) and toileting (*p* = 0.007) among the dependent special needs students were the significant factors of MSD among the special education teachers [26]. Moreover, frequently carrying and lifting heavy loads of more than 20 kg was two times more likely to cause MSD among special education teachers in Germany (AOR = 2.69, 95% CI = 1.53–4.75) [28]. This is not unexpected, given that working in special schools entails all-day care for students aged 6 to 17 years, with some dependence [11].

### 5.3. Different Body Areas

Different physical risk factors can cause MSD in different areas of the body. This has been proven by H. K. Cheng et al., where assist feeding has caused MSD in the shoulder area twice as much as any other task (OR = 2.077, 95% CI = 1.125–3.833) [11]. While performing these actions, the shoulder offers stability, mobility, or both. At the same time, teachers would need to regulate the kid’s bobbing head (shoulder acting as a stabilizer) and feed the child (shoulder moving). In addition, teachers may need to massage a kid’s chewing muscles and avoid aggravating their spastic mouth movements if they have weak oral mobility, such as reflexive biting [11].

Meanwhile, assisted toileting (OR = 2.842, 95% CI = 1.511–5.346) and rehabilitation (OR = 2.144, 95% CI = 1.123–4.095) were reported to cause MSD twice, likely in the lower back region [11]. To suit the children’s height or the size of the furniture, teachers must bend their waist and knees. Constant bending with muscle exertion is harmful to the lower back. Similar issues arise while assisting with toileting since bathroom settings are not meant for adults, such as commode sizes. According to a study conducted in Germany, special education teachers who were involved in nursing care, such as carrying, lifting, and transferring pupils (OR = 1.70, 95% CI = 0.96–3.00), washing pupils (OR = 1.93, 95% CI = 1.24–3.01), providing toilet assistance (OR = 2.03, 95% CI = 1.05–3.91), changing diapers (OR = 2.07, 95% CI = 1.20–3.56), and helping in dressing pupils (OR = 2.79, 95% CI = 1.29–6.06), had a significantly higher prevalence of chronic back pain up to two-fold [28]. Furthermore, a study in Japan revealed that teachers who assisted with mobility, excretory function, eating, skincare, and bathing had a significantly higher prevalence of MSD in the lower back [27].

## 6. Effect of Psychological Factors on MSD

As they lead their classes and create learning opportunities for their students, teachers continuously navigate complicated social conditions in the classroom. Teachers’ work-related stress has grown dramatically over the previous decade, resulting in one of the highest rates of burnout, with many teachers quitting or retiring early [35,36]. On the other hand, teachers experience greater tiredness, weariness, headaches, and tension than other professionals [37]. Therefore, psychological stress is another aspect to consider in addition to the factors associated with MSD. Zamri et al. reported that depression (OR = 1.98, 95% CI = 1.67–2.36), anxiety (OR = 1.73, 95% CI = 1.52–1.97), and stress (OR = 1.49, 95% CI = 1.11–1.99) symptoms were shown to strongly correlate with MSD among the teachers [14].

Teacher stress is characterized as unpleasant, negative feelings, such as anger, irritation, worry, despair, and nervousness, experienced by teachers due to some part of their work. These conditions could be seen in a study in Selangor where pain catastrophizing (OR = 2.27, 95% CI = 1.47–3.51), fear-avoidance belief towards physical activity (OR = 1.66, 95% CI = 1.18–2.34) and towards their job (OR = 1.79, 95% CI = 1.26–2.53), severe anxiety (OR = 2.87, 95% CI = 1.90–4.32), depression symptoms (OR = 2.58, 95% CI = 1.01–6.69), and somatizing tendency (OR = 2.13, 95% CI = 1.55–2.93) were all likely to cause MSD among teachers [15]. Teachers may be anxious due to challenges in the educational system and demands from schools, students, parents, and the community. Teachers experience significant psychological stress due to high job expectations, limited work control, and inadequate social support [38].

It has been discovered that special education teachers have a high degree of perceived stress. In the United States, special education teachers were shown to have a high score on the Perceived Stress Scale and Professional Quality of Life, reflecting the stress and fatigue faced by the teachers [39]. This might be since special education teachers are expected to meet the specific requirements of their students, along with the stress of dealing with special students’ learning challenges and violent conduct, which exacerbates special education teachers’ predicament. A study among special education teachers in Nigeria reported that about four in every ten teachers had psychological distress, representing many-fold the rates reported in the general population [40]. Furthermore, 26.8% of teachers reported mild stress, 8.9% moderate depression, and 2.8% moderately severe or severe depression in a study in Oregon, USA [41].

## 7. Conclusions

The findings of this review clearly show that regular and special education teachers are at risk of developing MSD. MSD is prevalent among the teachers and most commonly affects the shoulder, lower back, neck, and wrist. A growing body of evidence shows that MSD is significantly associated with the teachers’ individual, physical, and psychosocial factors. Individual factors related to MSD include age, duration of teaching experience, working hours, nap time, teaching burden, and partner. Physical factors, such as awkward posture, frequent lifting, carrying, transferring, toileting, changing diapers, rehabilitation, and feeding, have been proven to increase the risk of MSD. Perceived stress, fatigue, psychological distress, and depression have been identified as psychosocial risk factors. Therefore, appropriate interventions, such as ergonomically designed workplaces, comprehensive ergonomic training, psychological approaches, and functional training, can be adopted. Additionally, further research, especially longitudinal research, is needed to better understand MSD among regular and special education teachers, focusing on ergonomic factors and multifaceted interventions. This would be a significant step forward in preventing MSD among the teachers.

In this review, several limitations were identified. First, there are not many papers, especially about MSD among special education teachers. Only the site of the MSD is primarily mentioned, not the type of MSD. Since many researchers relied on anonymous surveys to acquire their data, recall bias and self-reporting could be seen as limitations in their research. Therefore, clinical diagnosis of MSD and its severity could be included in future studies, ideally with a longitudinal design. Future studies should also use a mixed-methods approach, including a more exacting quantitative approach such as observational studies, which involve physically observing teachers perform their jobs and checking their workplaces for further risk factor identification.

## Figures and Tables

**Table 1:** Studies reporting the prevalence of MSD among regular school teachers.

Study	Location	Year	Study Population	Sample Size	Body Site	Prevalence of MSD (%)
Ceballos & Santos	Pernambuco, Brazil	2015	Kindergarten and elementary grades teachers	525	Shoulders, upper back, neck, ankle/feet	73.5
Chaiklieng & Suggaravetsiri	Nakhonphanom, Thailand	2012	Primary and secondary school teachers	718	Lower back, shoulder, upper back, neck, arm	73.7
Ebied	Cairo, Egypt	2015	Primary school teachers	200	Lower back, neck, shoulder	76
Mohd Anuar et al.	Putrajaya, Malaysia	2016	Secondary school teachers	120	Lower back	72.9
P. N. Erick & Smith	Botswana, Africa	2014	Primary and secondary school teachers	1747	Lower back	55.7
Temesgen et al.	Gondar Town, Northwest Ethiopia	2019	Primary and secondary school teachers	754	Shoulder and neck	57.3
Yue et al.	Guangdong Province, China	2012	Primary, junior middle, and secondary school teachers	893	Neck, shoulder, lower back	48.7

**Table 2:** Studies reporting the prevalence of MSD among special education school teachers.

Study	Location	Year	Study Population	Sample Size	Body Site	Prevalence of MSD (%)
Atikah et al.	Kelantan, Malaysia	2019	Special education teachers	103	Lower back, shoulder, neck	72
Claus, Matthias et al.	Rhineland-Palatinate, Germany	2014	Special education teachers and educational staffs	395	Lower back	38.7
H. Y. K. Cheng et al.	Taiwan (multiple regions)	2013	Special education teachers	323	Shoulder, lower back, neck	94
H. Y. K. Cheng et al.	Taiwan (multiple regions)	2016	Special education teachers and teacher’s aides	388	Lower back, shoulder, wrist, knee	86
Muto, Shigeki et al.	Shizuoka, Japan	2006	Special education teachers and assistant staffs	975	Lower back	45
Pillastrini et al.	Forli, Italy	2009	Special education teachers	71	Neck, lower back	85.9
Wong et al.	Hong Kong	2009	Special education teachers, teaching assistants, and health care professionals	33	Lower back	55

## Data Availability

Not applicable.
